# Osteointegration of functionalised high-performance oxide ceramics: imaging from micro-computed tomography

**DOI:** 10.1186/s13018-024-04918-2

**Published:** 2024-07-18

**Authors:** Filippo Migliorini, Jörg Eschweiler, Marcel Betsch, Nicola Maffulli, Markus Tingart, Frank Hildebrand, Sophie Lecouturier, Björn Rath, Hanno Schenker

**Affiliations:** 1 Department of Orthopaedic, Trauma, and Reconstructive Surgery, RWTH University Hospital, Pauwelsstraße 30, 52074 Aachen, Germany; 2 Department of Orthopaedic and Trauma Surgery, Academic Hospital of Bolzano (SABES-ASDAA), 39100 Bolzano, Italy; 3 Department of Life Sciences, Health, and Health Professions, Link Campus University, Rome, Italy; 4 Department of Orthopaedic and Trauma Surgery, University Hospital of Erlangen, 91054 Erlangen, Germany; 5 Department of Trauma and Orthopaedic Surgery, Faculty of Medicine and Psychology, University La Sapienza, 00185 Rome, Italy; 6 Faculty of Medicine, School of Pharmacy and Bioengineering, Keele University, ST4 7QB Stoke On Trent, England; 7 Queen Mary University of London, Barts and the London School of Medicine and Dentistry, Mile End Hospital, 275 Bancroft Road, E1 4DG London, England; 8 Ortho-Centrum Aachen (OCA), Aachen, Germany; 9 Department of Orthopaedic Surgery, Klinikum Wels-Grieskirchen, 4600 Wels, Austria

**Keywords:** Ossification, Implants, Osteointegration, Micro-CT

## Abstract

**Background:**

This study evaluated the osseointegration potential of functionalised high-performance oxide ceramics (HPOC) in isolation or coated with BMP-2 or RGD peptides in 36 New Zeeland female rabbits using micro-computed tomography (micro CT). The primary outcomes of interest were to assess the amount of ossification evaluating the improvement in the bone volume/ total volume (BV/TV) ratio and trabecular thickness at 6 and 12 weeks. The second outcome of interest was to investigate possible differences in osteointegration between the functionalised silanised HPOC in isolation or coated with Bone Morphogenetic Protein 2 (BMP-2) or RGD peptides.

**Methods:**

36 adult female New Zealand white rabbits with a minimum weight of three kg were used. One-third of HPOCs were functionalised with silicon suboxide (SiOx), a third with BMP-2 (sHPOC-BMP2), and another third with RGD (sHPOC-RGD). All samples were scanned with a high-resolution micro CT (U-CTHR, MILabs B.V., Houten, The Netherlands) with a reconstructed voxel resolution of 10 µm. MicroCT scans were reconstructed in three planes and processed using Imalytics Preclinical version 2.1 (Gremse-IT GmbH, Aachen, Germany) software. The total volume (TV), bone volume (BV) and ratio BV/TV were calculated within the coating area.

**Results:**

BV/TV increased significantly from 6 to 12 weeks in all HPOCs: silanised (P = 0.01), BMP-2 (P < 0.0001), and RGD (P < 0.0001) groups. At 12 weeks, the BMP-2 groups demonstrated greater ossification in the RGD (P < 0.0001) and silanised (P = 0.008) groups. Trabecular thickness increased significantly from 6 to 12 weeks (P < 0.0001). At 12 weeks, BMP-2 promoted greater trabecular thickness compared to the silanised group (P = 0.07), although no difference was found with the RGD (P = 0.1) group.

**Conclusion:**

Sinalised HPOC in isolation or functionalised with BMP-2 or RGD promotes in vivo osteointegration. The sinalised HOPC functionalised with BMP-2 demonstrated the greatest osseointegration.

## Introduction

The number of joint arthroplasties is increasing and the debate over implant fixation and bearing surfaces is still ongoing [1, 2]. Also, the number of revision arthroplasties has increased. Component wear and tear and aseptic loosening are two major causes of revision [1, 3]. Aseptic loosening is the cause of revision arthroplasty in approximately 10% of patients in the US [4]. The persistent mechanical load can lead to implant wear and generate micro- and nano-particles, which can cause an inflammatory reaction resulting in bone resorption**.** Different materials have been manufactured to minimize component wear and tear and implant loosening. Ceramics implants are associated with a low incidence of biologically active particle generation and osteolysis. However, the low fracture toughness and linear elastic behaviour of ceramic implants make them prone to breakage under stress. To overcome these limitations, high-performance oxide ceramics (HPOC) have been introduced [5, 6]. HPOC implants show lower wear and biological response to debris [7]. Nevertheless, as HPOCs are biologically inert, implant osseointegration may be impaired [8, 9]. To overcome this limitation to clinical application, we developed biologically functionalised HPOC [10, 11]. Three different coating modalities were evaluated: (1) isolated silanised HPOC (sHPOC) with or without (2) Arg-Gly-Asp (sHPOC-RGD) or (3) bone morphogenic protein-2 (sHPOC-BMP2) peptide coatings [12–14]. For each coating method, we evaluated contact guidance, adhesions, surrounding mesenchymal stromal cells osteogenic differentiation, and their cytotoxic potential [15, 16]. Moreover, the osseointegration potential of functionalised HPOC was compared with titanium implants in rabbits using histomorphometry [12–14]. All coating modalities similarly promoted in vivo ossification to the titanium implants [12–14].

This study evaluated the osseointegration potential of functionalised sHPOC in isolation or coated with BMP-2 or RGD peptides in 36 New Zeeland female rabbits using micro-computed tomography (micro-CT). The primary outcomes of interest were to assess the amount of ossification evaluating the improvement in the bone volume/ total volume (BV/TV) ratio and the trabecular thickness at 6 and 12 weeks. The second outcome of interest was to investigate possible differences in osteointegration between the functionalised sHPOC in isolation or coated with BMP-2 or RGD peptides. It was hypothesised that functionalised sHPOC coated with BMP-2 or RGD peptides promotes greater osseointegration.

## Methods

### Sample preparation

The HPOCs used in all experiments were manufactured at the Department of Materials Science and Biomaterial Research of the RWTH University Aachen, Germany, as reported in greater detail previously [15, 17–23]. Briefly, plasma-enhanced chemical vapour deposition (PE-CVD) was used to pair stable organosilane monolayers on the monolithic Al_2_O_3_ HPOC-based cylinders. HPOC cylinders were functionalised with silicon suboxide (SiOx), which was deposited on the polished cylinders. HPOC were air-dried, cured at 80° for 45 min, and stored in liquid nitrogen until use. One day before the experiments, one-third of the sHPOCs were coated with BMP-2 (sHPOC-BMP2), and another third with RGD (sHPOC-RGD) [12, 13, 15, 18].

### Surgical procedure

**Fig. 1:**
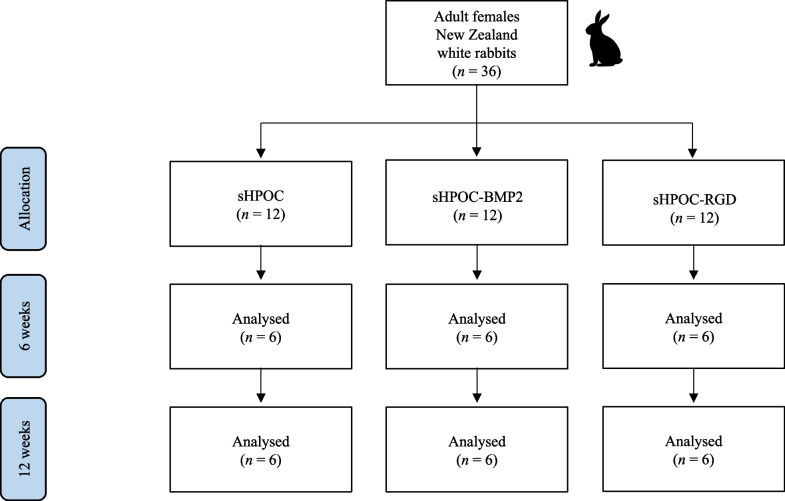
Study set up

This study was conducted according to the Animal Welfare Act of the Federal Republic of Germany. This study was approved by the Federal Office for Nature, Environment and Consumer Protection of North Rhine-Westphalia, Federal Republic of Germany (Approval ID: 84–02.04.2016.A434). The investigation involved 36 adult female New Zealand white rabbits with a minimum weight of three kg. Rabbits were randomly divided into three groups (Fig. 1): sHPOCs, sHPOC-BMP2, and sHPOC-RGD.

Before surgery, general anaesthesia was provided with 0,1 ml/mg/kg bodyweight Medetomidin hydrochloride (Domitor, Vetoquinol GmbH, Ismaning, Germany) combined with 0.2 ml Ketamin hydrochloride 10% (Narketan, Vetoquinol GmbH, Ismaning, Germany) via subcutaneous injection. The surgical site was shaved, disinfected with iodine and ethanol, and draped in a sterile fashion. Before incision, 10 mg/kg body weight of Enrofloxacin (Baytril, Bayer Austria GmbH, Wien, Austria) was injected subcutaneously. A longitudinal skin incision was performed over the right lateral femoral condyle. After accurate dissection through the fascia and muscles, the condyle was exposed. The lateral collateral ligament (LCL) was identified as a landmark. Sparing the LCL, a mono-cortical drill hole with a 5.5 mm trephine was prepared, and the HPOC cylinder was inserted in a press-fit fashion. Attention was given not to damage the knee capsule. After irrigation with saline solution, tissues were closed in layers. Finally, the skin was stapled and sealed with a chelated silver spray. For the first three days after surgery, 4 mg/kg bodyweight Carprofen (Rimadyl, Zoetis Deutschland GmbH, Berlin, Germany) was applied every 24 h. At six and 12 weeks postoperatively, the animals were euthanised with 2 ml/kg bodyweight natrium Pentobarbital (Fagron GmbH & Co. KG, Glinde, Germany), and the femoral condyles were harvested en bloc. Fixation was performed over 12 days with 4% paraformaldehyde followed by an alcohol series with ethanol of 50% to 100% and xylol. The specimens were embedded in Technovit® 9100 (Heraeus Kulzer GmbH, Hanau, Germany).

### MicroCT evaluation

**Fig. 2:**
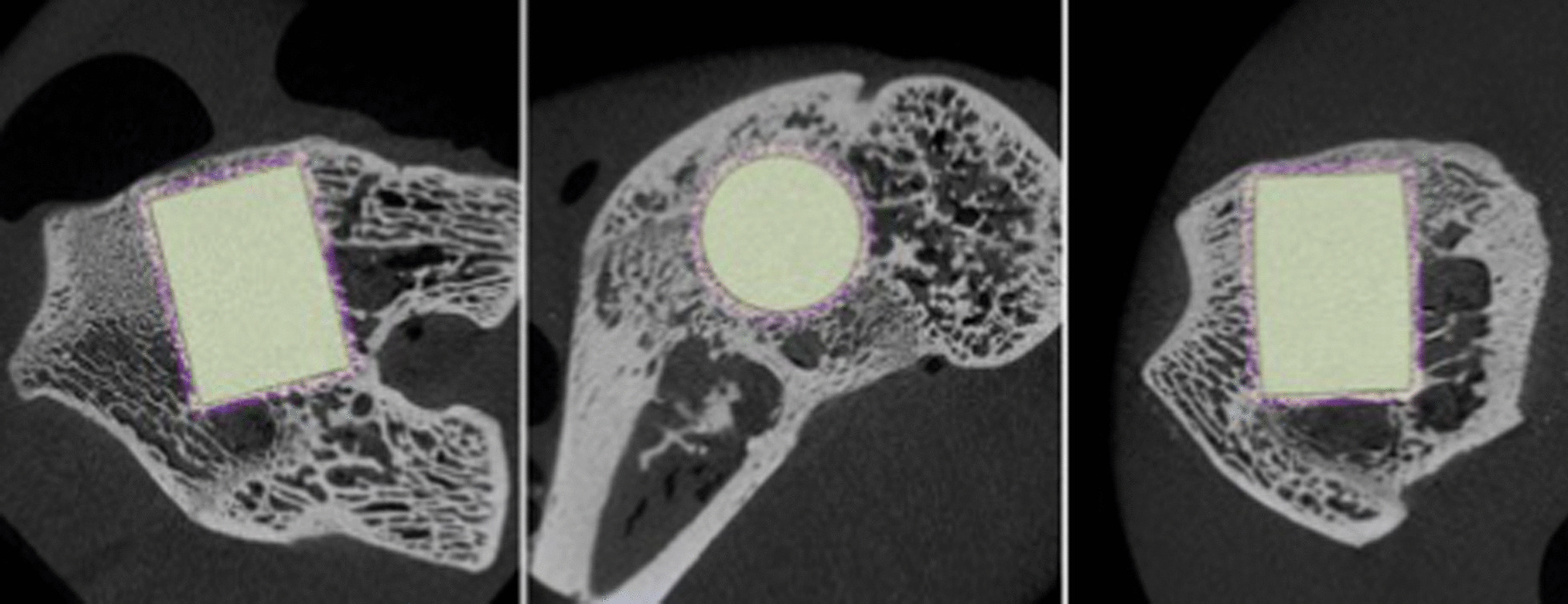
Three-dimensional MicroCT sequences of the condyle and implants. The interface bone implant (region of interest, ROI) is highlighted around the implant

After PMMA embedding, all samples were scanned with a high-resolution micro CT (U-CT^HR^, MILabs B.V., Houten, The Netherlands) with a reconstructed voxel resolution of 10 µm. MicroCT scans were reconstructed in three planes and processed using Imalytics Preclinical version 2.1 (Gremse-IT GmbH, Aachen, Germany) software (Fig. 2).

**Fig. 3:**
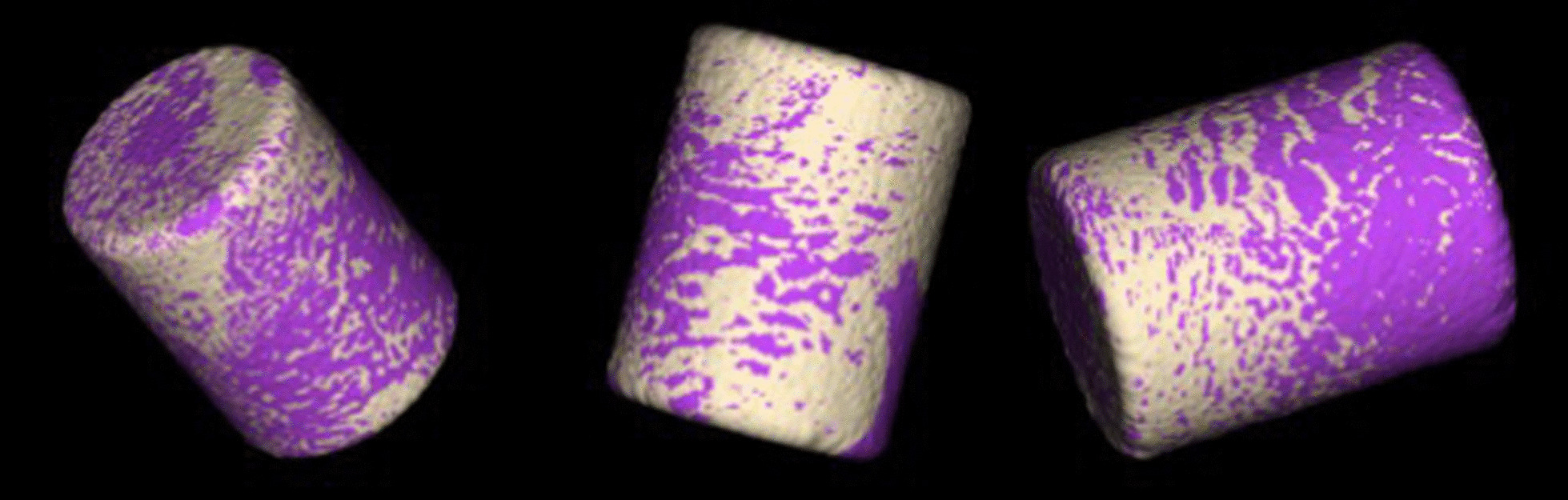
Three-dimensional Surface Implant after thresholding (White = Bony tissue)

To eliminate any surrounding soft tissue and epoxy a primary density thresholding with a predefined value of 5000 was performed. To gain smoother edges an anti-aliasing was performed manually with 3 voxels. Subsequently, an ROI (coating area) was defined with a value of 10 (Fig. 2). To differentiate between bone and soft tissue another density thresholding with a predefined value of 3000 within the ROI was performed. The bony tissues were marked in white, whereas the surrounding space within the ROI was marked purple. For better visualization and further calculation, a three-dimensional model was calculated (Fig. 3).

**Fig. 4:**
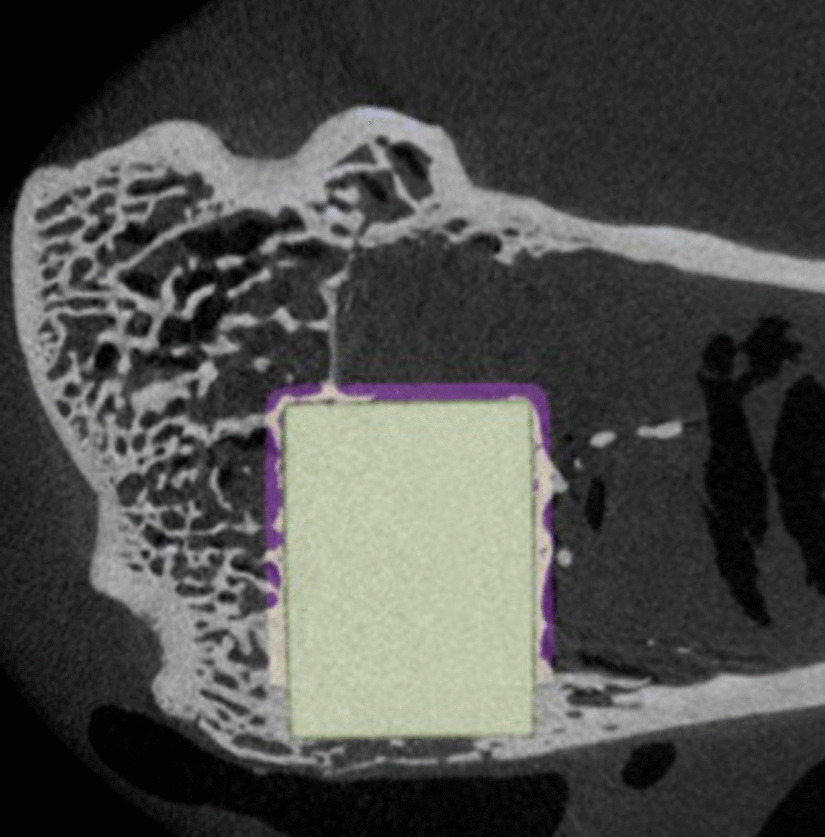
Two-dimensional MicroCT sequence showing the cropped area (lateral deleted ROI) and showing the surgical defect area on the lateral aspect of the implant

**Fig. 5:**
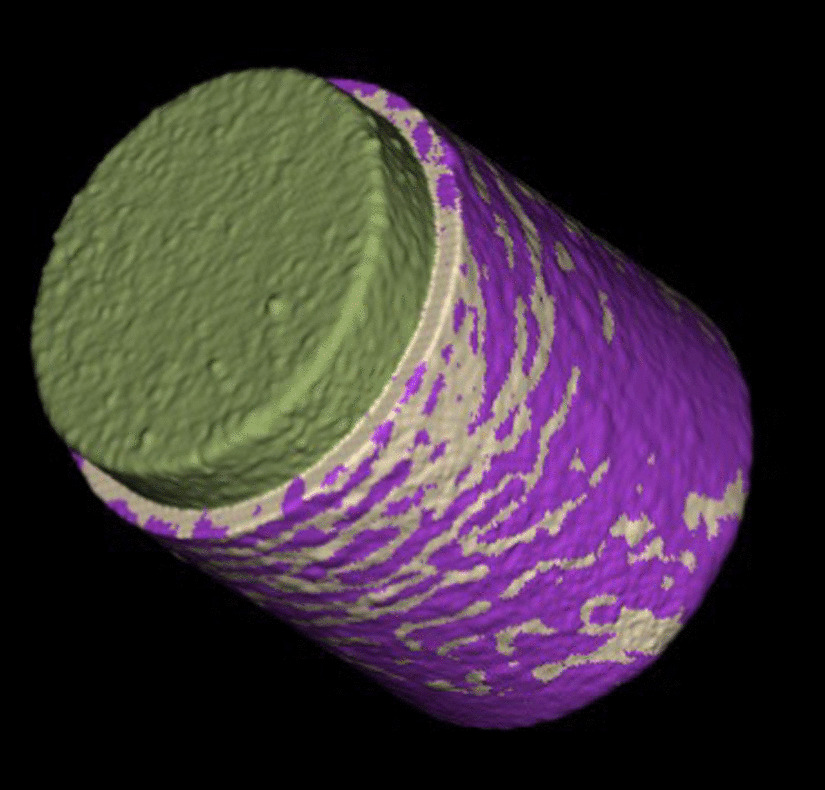
Three-dimensional model showing the cropped area, bony tissue in white and soft tissue in purple

To eliminate the bias caused by the bony approach on the lateral aspect of the implant within the ROI, a predefined crop was performed in all samples (Fig. 4). The cropped region was not considered for further calculation. After performing the cropping, a three-dimensional model was calculated for better visualization and following measures (Fig. 5).

The total volume (TV) of the ROI was determined. Within the TV, the bone volume (BV) was evaluated, and the ratio BV/TV was calculated using an implemented feature of the Imalytics software. Finally, the trabecular thickness was measured using an implemented tool of the Imalytics software. Data were extracted to Microsoft Office Excel version 2016 (Microsoft Corporation, Redmond, Washington, USA).

### Outcomes of interests

The outcomes of interest were to evaluate the BV/TV ratio and the trabecular thickness (µm) of each group and compare them at 6 and 12 weeks of follow-up.

### Statistical analysis

The statistical analyses were conducted by one author (**) using the software STATA / MP, version 14.1 (Stata Corporation, College Station, Texas, USA). To investigate whether the within-group BV/TV ratio and the trabecular thickness improved between 6 and 12 weeks, the mean difference (MD) effect measure and standard error (SE) were used. The paired two-tailed t-test was performed.

To compare the between-group BV/TV ratio and the trabecular thickness at 6- and 12 weeks, analysis of variance (ANOVA) was used. The variance (VAR), the sum of squares (SS), and the Fisher-Snedecor distribution (F) were analysed. If P_ANOVA_ < 0.05, the Tukey Honestly Significant Difference (HSD) posthoc test was performed to investigate each direct comparison. Confidence intervals were set at 95%. Values of P < 0.05 were considered statistically significant.

## Results

### Animal characteristics

34 rabbits survived the 6- or 12-week experimental period. One rabbit in the RGD group and one in the BMP-2 group perished. Five wounds dehiscence were stapled. At euthanasia, no clinical signs of inflammation or adverse tissue reactions were observed. All implants remained in situ. The mean weight of the rabbits increased from baseline to the last follow-up of + 501.3 mg.

### Evaluation of BV/TV ratio

Within-group BV/TV ratio increased significantly from 6 to 12 weeks in all experiments (Table 1): isolated silanised HPOC (MD 2.21; P = 0.01), silanised HPOC coated BMP-2 (MD 5.53; P < 0.0001), and coated RGD (MD 1.03; P < 0.0001). The ANOVA test showed no between-group difference at 6 (P = 0.06) and 12 weeks (P = 0.1).

**Table 1:** BV/TV at 6 and 12 weeks (MD: mean difference; SE: standard error; CI: confidence interval)

Endpoint	6 weeks	12 weeks	MD	SE	95% CI	*P*
Silanised	8.7 ± 2.0	10.9 ± 3.1	2.2	0.821	3.87 to 0.54	0.01
BMP-2	4.7 ± 1.2	10.2 ± 2.4	5.5	0.598	6.74 to 4.31	< 0.0001
RGD	6.3 ± 1.6	9.7 ± 2.1	3.4	0.579	4.57 to 2.22	< 0.0001

### Evaluation of trabecular thickness

Within-group trabecular thickness (µm) increased significantly from 6 to 12 weeks in all experiments (Table 2): silanised HPOC (MD 1.92; P < 0.0001), silanised HPOC coated BMP-2 (MD 2.20; P < 0.0001), and coated RGD (MD 3.15; P < 0.0001). The ANOVA test showed no between-group difference at 6 (P = 0.1) and 12 weeks (P = 0.1).

**Table 2:** Trabecular thickness (µm) at 6 and 12 weeks (MD: mean difference; SE: standard error; CI: confidence interval)

Endpoint	6 weeks	12 weeks	MD	SE	95% CI	*P*
Silanised	5.67 ± 0.46	7.59 ± 1.04	1.92	0.251	1.40 to 2.43	< 0.0001
BMP-2	5.55 ± 0.77	8.00 ± 1.20	2.45	0.319	1.80 to 3.09	< 0.0001
RGD	5.55 ± 0.47	8.70 ± 1.00	3.15	0.247	2.64 to 3.65	< 0.0001

## Discussion

According to the main findings of the present study, silanised HOPCs in isolation or functionalised with BMP-2 or RDG peptide promoted similar osteointegration in vivo. BV/TV ratio and trabecular thickness increased significantly from 6 to 12 weeks in all HPOCs. However, we were unable to demonstrate a statistically significant difference between the three groups.

Although ceramic is bioinert, its material topography, such as roughness and surface structure, influences cellular response [24, 25]. The microcapillary array structures on the alumina ceramics promoted mesenchymal cell migration, and bone mineralisation [16]. Over the past years, many inorganic materials have been employed to overcome the problem of ceramic bio inertia such as hydroxyapatite and bioactive glasses [26, 27]. Böke et al. [15] showed an increment of cell adhesion using an interfacial layer of silicon suboxide. PE-CVD enhanced the interfacial bond strength between the ceramic and silicon layer and produced high-degree cross-linking within the film [15]. Our previous study investigated the differences in osseointegration between HPOCs and titanium implants. A greater osteoid implant contact in the HPOCs group than in the titanium group after 6 weeks was evident [14]. The silica layer promotes protein absorption that facilitates cell attachment on the implant’s surface [28].

BMP-2 promotes osteoblast proliferation, stimulates mineralisation, and enhances mRNA and protein expression of the main osteogenic inductors [29]. BMP-2-enhanced scaffolds have been employed to treat bone defects, inducing significant bone regeneration [30, 31]. High BMP-2 concentration can activate the osteolytic pathway [32]. Hunziker et al. [33] conducted an animal study on 18 sheep comparing titanium implants coated with different concentrations of BMP-2 versus control non-coated implants. After 3 weeks the implants with the highest dose of BMP-2 showed a bone-implant contact ratio (BIC) decrement compared to the control group. The rest of the BMP-2 implants showed an increment in BIC compared to the control group. We previously compared the osseointegration of HPOCs functionalised with BMP-2 implants and titanium implants in 36 rabbits [12]. BIC was higher in the BMP-2 group than in the control group after 6 and 12 weeks. Osteoid implant contact was higher in the BMP-2 group than in the control group after 6 weeks, with no statistically significant difference after 12 weeks.

RGD peptide mediates integrin-specific cell adhesion [34]. RGD-integrin complex binds mesenchymal cells and activates intracellular signalling by different kinds of pathways that lead to the activation of RUNX2 and osteocalcin [34, 35]. It promotes mesenchymal cell migration, osteoblast differentiation and osseointegration [36]. Rappe et al. [37] compared RGD-coated titanium implants, thermally bioactivated titanium implants and titanium implants in 18 rabbits. No statistically significant difference in BIC was found between the three groups after 4 and 12 weeks. Quantitatively comparing the three groups, RGD implants showed the highest BIC. In our previous study on 36 rabbits, the differences between HPOCs functionalised with RGD implants and titanium implants were analysed [12]. The RGD group showed higher BIC than the titanium group after 6 and 12 weeks. No statistically significant difference existed between the two groups after 12 weeks.

The stability of a prosthetic implant is crucial to avoid aseptic loosening and implant failure [38, 39]. Final implant stability is reached in two steps [40]. Primary stability depends on the relative micromovement between the bone and the implant induced by the physiological joint loading [41]. Implant positioning, surgical technique, and bone quality can influence primary stability [42]. Secondary stability is given by the osseointegration process [43]. Osseointegration guarantees the proper anchorage of the implant to the bony tissue [44]. The first radiographic signs of osseointegration are visible three months after surgery [45]. The process of osseointegration of HPCO implants is faster than titanium implants [12, 14]. A meta-analysis on 21,000 total knee arthroplasties demonstrated a statistically significant association between early migration of tibial components and late revision for aseptic loosening [46]. Strait et al. [47] investigated the early mobilisation of the implant in 158 cementless THA. Early mobilisation, up to two years postoperatively was a risk factor for aseptic loosening. The faster secondary stability is reached, the lower the rate of early mobilisation of the implant. BMP-2 implants showed the highest osseointegration potential after 12 weeks [47], but longer follow-up studies are needed.

Our results confirm that biological augmentation of ceramic implants promotes osseointegration. At present, few studies analyse the in vivo interactions between bone tissue and HPOC implants, and our results must be confirmed by further studies on a larger population with longer follow-up.

This study has some limitations. Firstly, animal models do not fully translate to human models. Differences exist in biological processes, functional anatomy, and mechanical loads that the human body must tolerate. All these can significantly affect the osseointegration process. Nevertheless, the rabbit model is reproducible, easy to handle, and cost-effective. The stability of the implant was not tested biomechanically, and the biomechanical characteristics of the newly formed bone were not investigated. The follow-up was limited to 6 and 12 weeks. According to the ANOVA, at 6 weeks all three groups were similar in BV/TV and trabecular thickness; however, small variability was evident, and whether this might influence the results at 12 weeks is unknown. Moreover, understanding the progression of new cancellous bone formation after 12 weeks on these functionalised HPOC surfaces can be of clinical benefit. Future studies should compare the performance of these functionalised HPOCs with standard materials used in arthroplasty (e.g. titanium).

## Conclusion

Sinalised HPOC in isolation or functionalised with BMP-2 or RGD promote in vivo osteointegration. The sinalised HOPC functionalised with BMP-2 demonstrated the greatest osseointegration.

## Data Availability

The data presented in this study are available on request from the corresponding author.

## Abbreviations

micro CT: Micro-computed tomography
HPOC: High-performance oxide ceramics
BMP-2: Bone Morphogenetic Protein 2
SiOx: Silicon suboxide
TV: Total volume
BV: Bone volume
PE-CVD: Plasma-enhanced chemical vapour deposition
LCL: Lateral collateral ligament
MD: Mean difference
SE: Effect measure and standard error
ANOVA: Analysis of variance
VAR: Variance
SS: Sum of squares
HSD: Honestly Significant Difference
BIC: Bone-implant contact ratio
